# Physical Trauma among Refugees: Comparison between Refugees and Local Population Who Were Admitted to Emergency Department—Experience of a State Hospital in Syrian Border District

**DOI:** 10.1155/2017/8626275

**Published:** 2017-06-14

**Authors:** Yigit Duzkoylu, Salim Ilksen Basceken, Emrullah Cem Kesilmez

**Affiliations:** ^1^General Surgery Department, Islahiye State Hospital, Gaziantep, Turkey; ^2^Neurosurgery Department, Islahiye State Hospital, Gaziantep, Turkey

## Abstract

**Background:**

Hundreds of thousands of people have fled to Turkey since the civil war started in Syria in 2011. Refugees and local residents have been facing various challenges such as sociocultural and economic ones and access to health services. Trauma exposure is one of the most important and underestimated health problems of refugees settling in camps.

**Aims:**

We aimed to evaluate refugee admissions to emergency department because of trauma in means of demographics of patients and mechanism of trauma and compare the results with the local population.

**Methods:**

Retrospective evaluation of results and comparison with the results of local population.

**Results:**

We determined that the ratio of emergency admission of refugee patients because of trauma was significantly higher than the local population for most types of trauma.

**Conclusion:**

Further studies with more refugee participants are needed to fully understand the underlying reasons for this high ratio to protect refugees as well as for planning to take caution to attenuate the burden on healthcare systems.

## 1. Introduction

Since the civil war started in Syria in March 2011, all of the neighboring countries have been influenced from the immigration of thousands of people. Many of Syrian and North Iraqi refugees have fled to Turkey in 5 years. More than 270.000 refugees are being hosted in refugee camps and nearly 50.000 of them in the province of Gaziantep. In spite of these compelling conditions, refugee camps in Turkey have been reported to be sufficient in terms of providing the basic needs of seekers according to the Helsinki Citizens Assembly (HCA) following various reports of international visiting delegations [1]. Trauma exposure is one of the most important and underestimated health problems of refugees settling in camps and we aimed to evaluate refugee admissions to emergency department because of trauma and compared our results with the local population.

## 2. Methods

The study took place in a state hospital which is the primary referral center for two main, official refugee camps in Gaziantep province, 713 kilometers far from Ankara the capital. The camps hosted to 27,191 refugees (18,324 Syrians and 8,867 Iraqis) at the time of study in 2015 [2]. The refugees receive basic healthcare at the camps; they are referred to hospitals for initial healthcare, using ambulance service for emergent cases and referred to city center for further research if necessary. Population of the region was reported to be 65,869 (excluding refugees) due to official reports [3].

Refugee patients that had been referred to emergency department between January 1st and December 31st in 2015 were evaluated in means of demographics and trauma mechanism. Later the results were compared with the local population within the referrals between the same period of time.

Statistical analysis was performed using SPSS 15.0 Chicago, USA. Data evaluation was made due to descriptive statistical methods (mean, standard deviation, and median) and Pearson Chi-square test for the qualitative data between groups. Statistical significance was determined with “*p*” value under 0,05 and in 95% confidence interval.

## 3. Results

Trauma types and distribution among refugee and local patients are shown in Table 1. The frequency of refugees admitted to the emergency department with trauma was statistically higher (*p* < 0,01) compared to the local population for most types of trauma and for overall trauma admissions. The frequency of assaults and motor vehicle accidents were found to be higher in the local population. Age and sex distribution among patients is shown in Table 2. As a result, we found out that females (*p* < 0.01) and patients under 18 years of age (*p* < 0.01) were significantly higher in refugee group.

## 4. Discussion

Hundreds of thousands of people have been forced to leave their homes and had to find refuge, medical, and social aid with the ongoing civil war in Syria since 2011. Turkey is in the middle of the biggest refugee problem of recent years. Similar to the others all over the world, refugees taking shelter in Turkey have to stand against various physical, psychological, and healthcare problems [4–8]. Total number of Syrians settling in Turkey by the time that the study had been conducted was reported to be over 2 million [9]. Registered Syrian refugees settling in camps were over 263 thousand [9]. On the same date, the number of registered Iraqi refugees was nearly 95 thousand [10]. There are totally 26 refugee camps in Turkey, in 10 different cities [2]. Two of them are settled in Islahiye district of Gaziantep province, containing over 18 thousand of Syrians and nearly 9 thousand Iraqis [10]. With the ongoing refugee flow to Turkey, asylum seekers come with a range of health problems not only emanating from prior diseases, but also resulting from their flight overseas or originating in camps.

Growing number of refugees is a heavy burden on the economy, especially for the cities that host refugee camps. Over the past 5 years, hundreds of thousands of refugees from especially 2 countries (Syria, Iraq) have been resettled in Turkey, first in tent cities and later in more developed refugee camps. As long as the civil war continues, refugees can not return back home, so their needs and problems will persist and increase. They present a significant and increasing challenge not only for economical and social statistics, but also for general practice for healthcare professionals, similar to some of the former European studies [11]. Although refugees come from different countries, their collective experiences allow for some suggestions to be made about general healthcare needs, challenges, and outcome expectations. Although there are studies in the literature that have evaluated posttraumatic stress disorder, various infectious diseases, and neonatal conditions among refugees [12–14], there is still limited published literature on healthcare systems among refugees who settle in Turkey.

Physical trauma is an entity that can be easily underrecognized in refugee camp conditions. In spite of the growing number of refugee population, there has not been conducted a detailed study describing the trauma among camp residents. Those people who have survived war, torture, and so forth continue to be exposed to underestimated high prevalence rates of trauma and related disorders. It may be very helpful and necessary for healthcare servers, especially surgeons and emergency doctors, to have sufficient data on this large group of patients.

In this retrospective study, we aimed to outline the prevalence of refugee admissions to emergency department because of trauma in means of demographics of patients and mechanism of trauma and compare the results with the local population. Although there are studies that have especially focused on posttraumatic stress disorder among refugees, we planned to accomplish a comprehensive epidemiological study in order to determine rates of physical trauma exposure among refugees in camps, which is one of the leading studies in this field.

In our results, we found that in refugees the prevalence of emergency department referrals because of trauma were highly significant when compared to the local population in most types of trauma: head injury, fracture, skin injuries, and burns. The lower prevalence of motor vehicle accidents likely reflects the limited presence of motor vehicles in camps and the greater number of people with a driving license and cars in the local population. Assault was found at a higher prevalence in the local population. This may be due to having an assault report for possible accusations in court for the future which may not be as useful for refugees living in camps with relatively limited access to police departments and the justice system. Although the number was not as high as expected, most of the head injuries were caused by assaults especially among young and male patients, but they had not been referred to the police department (male: 67,1%, under 18 years: 76,9%). Therefore, the excess prevalence of head injuries among the refugee patients may partially reflect a shift from what otherwise would be recorded as assaults.

Number of burns (including electricity shock) and mean age of patients were found to be prominently significant (mean age: 11.04 and 242 patients under 18 years of age) when compared to local population despite the limited presence of electrical tools and boiling water, which we think are important in means of a major defect in basic childcare. This consideration is also consistent with the statistically significant high percentages of females and under 18-year-old parents in the refugee group. Although burns can be involved among pediatric problems in local population especially in this part of the country, still the frequency in refugee children was significantly higher. We think that particular attention should be paid for the care of refugee children. Also the rate of births among refugees, in camps and health institutions, is much more higher than the rates of the country, which results in another burden on the economical status of health services and another challenge for healthcare professionals, which may be a difference from former European studies.

In their 10 years of evaluation in a Swiss emergency department, Pfortmueller et al. reported among 3,675 refugees that male patients were more and younger than females, significantly [15]. Similarly, Müller et al. determined more specifically that trauma was higher in males in means of emergency admittance in their study involving 1,667 patients in 3 years [16]. Our findings were also similar to those results in means of age and gender (Table 2). But the main difference was that we compared our results with the local population. Additionally, refugee admittance per day was much more in our hospital, depending on the fact that it was in a special region of the country nearby the Syrian border and the initial reference health center for two main refugee camps.

During the same time period, total annual admissions to emergency department were 177,686, consisting of 76,869 refugees and 100,817 patients of local public. When those numbers were divided by the population of the two groups, admission of local patients was 1.53 admissions per person in the local population while the corresponding ratio was 2.82 admissions per person in the refugee population. The high frequency of total admissions to the emergency department is indeed typical for this part of the country and may suggest a deficit of primary healthcare resources for the local population as well as refugees as it has been indicated in the former reports of Turkish Ministry of Health [17]. Leading causes of emergency admissions in both groups were acute gastroenteritis and upper respiratory tract infection in patients under 18 years old. Annual admissions due to trauma were 2,25% among emergency admission of refugees and 2,11% among local patients. In refugee patients, about 82% of the patients were treated as outpatients, 11% of them were interned to hospital, and about 7% of them were transferred to secondary hospitals in city center. These results show that trauma admissions are actually a relatively small fraction of overall emergency admissions (a little over 2% for both refugees and local population), but their possible prognoses such as surgical procedures, extensive operations, longer hospital stays, repeated radiological and laboratory studies, control examinations as outpatients, and leaving permanent damage in patients increase health costs which emphasize their importance rather than numerical values among other admissions.

These statistically significant differences between refugees and local population can be explained with the main problems of Syrian refugees such as low living standards, lack of adequate child care and hygiene, somatization of psychological disorders, and also the possible underlying relationship between posttraumatic stress disorder and physical violence which needs further evaluation. But the higher ratio of trauma exposure is not the only reason of these significant findings. Refugees usually tend to admit to emergency clinic for various reasons such as relatively easier accessibility of emergency clinic and abuse of health system to leave the camps more easily. It is a known fact that, in countries with good healthcare systems, patients are admitted to clinic for outpatients in working hours and can be referred to emergency clinics for 24 hours during the day in case of emergency. In our results, we determined a significantly higher ratio of emergency admissions for refugee patients not only for trauma, but also for other health problems when compared to local population. This issue is emphasized in more detail in “challenges” part.

With the exception of studies concerning infectious diseases and posttraumatic stress disorders, there are only a few clinical trials evaluating the health problems of refugees after arriving and settling in Turkey. Although it has not been mentioned particularly in the literature yet, trauma exposure is a serious health problem among refugees in camps, resulting from numerous reasons such as facing hard living conditions, living among a crowded group in a limited area, and working at hard labour jobs.

*Limitations and Challenges*

*Language*. Although there are translators in each department of the hospital and the camps, it can be still challenging for refugees to explain their complaints and medical history properly. Qualified translators are indispensable for caring about refugee patients.

*Social Awareness*. Many times refugees with chronic systemic diseases, congenital abnormalities, and disorders that have been present even before leaving their hometown refer to the emergency department, as it has been reported as an extra burden on the healthcare sector. This may be due to relatively faster accessibility of emergency when compared to outpatient clinics or the fact that refugees are let to leave the camp for emergent cases more easily, which results in abuse of the health system.

## 5. Conclusion

The ratio of emergency admission of refugee patients because of trauma is significantly higher than local population. Our results show the need for emergency doctors, surgeons, nurses, and social workers who work with refugee population, especially in regions that camps are settled in. Comprehensive planning, support, and registration of patients are required for a more wide-ranging understanding of the needs of refugee population with refining early preventative healthcare measures to avoid more serious diseases or traumas which will cause minimal burden on the local healthcare system. For the potential prevention of this workload on health institutions resulting in the insufficiency in the number of professionals or the facilities of secondary or tertiary hospitals, directing the resource allocation to the initial healthcare primarily in camps may be beneficial. Health education programs can help to adapt cultural differences. Because of the single-center design of this study, multicenter studies with larger refugee populations are necessary to fully evaluate the sociocultural and health problems of refugees, as well as their health beliefs.

## Figures and Tables

**Table 1:** Emergency referrals of refugee and Turkish patients (trauma).

Diagnosis	Refugee patients			Turkish patients			*p*; *χ*^2^
		Per 10000 patients	%^**∗**^		Per 10000 patients	%^**∗**^	
Head injury^1^	329	120.9	18.9	281	42.6	13.2	*p* < 0.05; 180.16
F, D, S^2^	639	235	36.8	122	18.5	5.7	*p* < 0.05; 1109.41
MVA^3^	57	20.9	3.2	279	42.3	13.1	*p* < 0.05; 23.90
Skin tear^4^	365	134.2	21	343	52	16.1	*p* < 0.05; 171.00
Assault^5^	45	16.5	2.5	648	98.3	30.4	*p* < 0.05; 173.24
Burn^6^	300	110.3	17.2	455	69	21.3	*p* < 0.05; 40.19
Total	**1735**	**638**	**100**	**2128**	**323**	**100**	**p** < 0.05**; 479.22**
Population	**27,191**	**10000**		**65,869**	**10000**		

^1^Head injuries involving fracture, bleeding, hematoma, closed head injury, superficial skin tear, and additional nose, mouth, and orbital injuries. ^2^Fracture, dislocation, sprain of extremities (including hand/foot finger amputations). ^3^Motor vehicle accident. ^4^Skin tears which need repair (except head). ^5^Physical assault, including shotgun. ^6^Including burns due to electric shock. ^**∗**^Percentage  in ER admission type.

**Table 2:** Demographics of patients.

	Refugee patients	Turkish patients	*p*; *χ*^2^
M (*n*, %)	1162 (66.9)	1508 (70.8)	*p* < 0.05, 6.60
F (*n*, %)	573 (33.0)	620 (29.1)	
<18 age (*n*, %)	1008 (58.0)	709 (33.3)	*p* < 0.05, 236.69
≥18 age (*n*, %)	727 (41.9)	1419 (66.6)	
Average (Age)	19.06	27.01	

M: male patients, F: female patients, <18: child patients, ≥18: adult patients, Turkish: local patients, refugee: patients from camps.
